# DNA DSB Repair Dynamics following Irradiation with Laser-Driven Protons at Ultra-High Dose Rates

**DOI:** 10.1038/s41598-019-40339-6

**Published:** 2019-03-14

**Authors:** F. Hanton, P. Chaudhary, D. Doria, D. Gwynne, C. Maiorino, C. Scullion, H. Ahmed, T. Marshall, K. Naughton, L. Romagnani, S. Kar, G. Schettino, P. McKenna, S. Botchway, D. R. Symes, P. P. Rajeev, K. M. Prise, M. Borghesi

**Affiliations:** 1 Centre for Plasma Physics, School of Mathematics and Physics, Queen’s University Belfast, Belfast, BT7 1NN UK; 2 Centre for Cancer Research & Cell Biology, Queen’s University Belfast, Belfast, BT9 7AE UK; 3 Extreme Light Infrastructure – Nuclear Physics (ELI-NP), Horia Hulubei Institute for Nuclear Physics (IFIN-HH), Reactorului Str., 30, Magurele Campus, Bucharest, Romania; 4 Laboratoire pour l’ Utilisation des Lasers Intenses (LULI), Ecole Polytechnique, 91128 Palaiseau Cedex, France; 5 National Physical Laboratory, Hampton Road, Teddington, TW11 0LW UK; 6 Department of Physics, SUPA, University of Strathclyde, Glasgow, G4 0NG UK; 7 Central Laser Facility, Science and Technology Facilities Council, Rutherford Appleton Laboratory, Harwell Campus, Didcot, OX11 0QX Oxford, UK

## Abstract

Protontherapy has emerged as more effective in the treatment of certain tumors than photon based therapies. However, significant capital and operational costs make protontherapy less accessible. This has stimulated interest in alternative proton delivery approaches, and in this context the use of laser-based technologies for the generation of ultra-high dose rate ion beams has been proposed as a prospective route. A better understanding of the radiobiological effects at ultra-high dose-rates is important for any future clinical adoption of this technology. In this study, we irradiated human skin fibroblasts-AG01522B cells with laser-accelerated protons at a dose rate of 10^9^ Gy/s, generated using the Gemini laser system at the Rutherford Appleton Laboratory, UK. We studied DNA double strand break (DSB) repair kinetics using the p53 binding protein-1(53BP1) foci formation assay and observed a close similarity in the 53BP1 foci repair kinetics in the cells irradiated with 225 kVp X-rays and ultra- high dose rate protons for the initial time points. At the microdosimetric scale, foci per cell per track values showed a good correlation between the laser and cyclotron-accelerated protons indicating similarity in the DNA DSB induction and repair, independent of the time duration over which the dose was delivered.

## Introduction

Several investigators have suggested^1–3^, the potential of laser-accelerated protons for future hadrontherapy applications. In this perspective, the development of compact laser based accelerators is currently motivating the activities of several significant research programmes worldwide^4^. Laser-driven ion acceleration technology is still evolving^5^ and a strong focus of these activities is on achieving the challenging developments in ion beam parameters, which will be required for translation of this technology to the clinics. In parallel, several groups have engaged in pre-clinical radiobiological experiments employing laser-accelerated ions^6–13^. These investigations have partly been aimed at establishing procedures for cell handling, irradiation and dosimetry, which are compatible with the complex laser-plasma interaction environment. Additionally, the radiobiological potential of employing such beams requires extensive investigation before they can then be utilized as a therapeutic tool. The main concern and drive behind the biological investigations is the large variation in beam parameters between conventional and laser based accelerators. In particular, the most significant difference is that the ion beams delivered from laser-driven accelerators are of an ultra-short pulse nature, as the ions are emitted in bursts of sub-picosecond duration from the laser source. The ion pulse duration then spreads in time during beam transport from the source to the target, typically delivering ion pulses in the nanosecond range at the irradiation site, depending on the energy selection implemented. The ultra-short dose deposition translates to an ultra high dose rate of the order of 10^9 ^Gy per second, many orders of magnitude higher than that of conventional ion beams (typically Gy/min). Under these conditions, effects related to the ultrashort dose deposition have been suggested as possible causes for variations in the biological response of the irradiated cell, namely through possible alteration of the indirect DNA damage associated to free radical production (oxygen depletion effect)^14^ or, at sufficiently high doses, spatio-temporal overlap of independent tracks resulting in collective effects^15^.

Radiobiological information at these ultra high dose rates is still limited, and experiments performed using laser-driven proton beams have not yet shown significant deviations (e.g. in terms of Relative Biological Effectiveness) from known biological responses with conventional beams at comparable LET (with the possible exception of some, even more limited, investigations of sub-lethal effects^12,16^). One should also note that in many of these experiments the required dose has been delivered in temporally spaced multiple fractions (e.g.^6–8,11,12^) so that, while the peak dose rate within a pulse is very high, the effective rate at which Gy-level doses are delivered becomes ‘comparable’ with established irradiation sources, which could in principle mask any potential effect associated to the highly pulsed deposition. Only three publications have so far reported Gy-level irradiation in single pulses, at ultra-high dose rates^8,9,15^, which is also the approach used here to study the DNA DSB damage and repair kinetics induced by single pulses of laser-accelerated 10 MeV protons at an ultra-high dose rate of 10^9^ Gy/s. We used a well-referenced radiobiologically relevant human cell line AG01522B^17–19^ and compared our results with lower LET X-rays and cyclotron accelerated protons.

## Results

### DNA DSB damage repair kinetics induced by laser-accelerated protons

**Figure 1:**
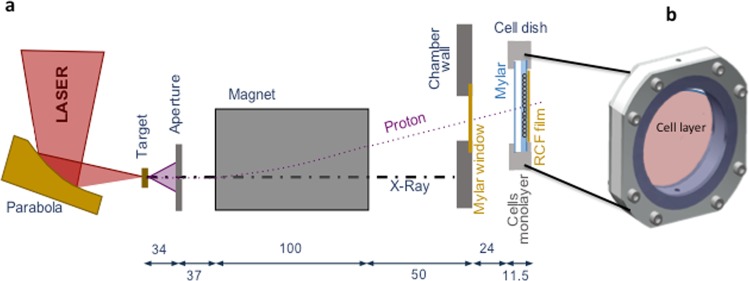
Experimental set up for irradiation of the AG01522 cells with 10 MeV laser accelerated protons at the Gemini Laser facility of the Rutherford Appleton Laboratory, Didcot, Oxford. (**a**) Schematic of the Laser interaction chamber and cell dish arrangement during irradiations (the distance is given in centimeters). (**b**) Design of the dish where cells were grown as monolayers on 3 µm thin Mylar. Before irradiation the dish was mounted with another piece of mylar, to prevent the drying of monolayers and the gap between the two mylar pieces was filled with cell culture medium. During irradiation the medium was withdrawn with a motorized syringe system and refilled after irradiation of cells.

**Figure 2:**
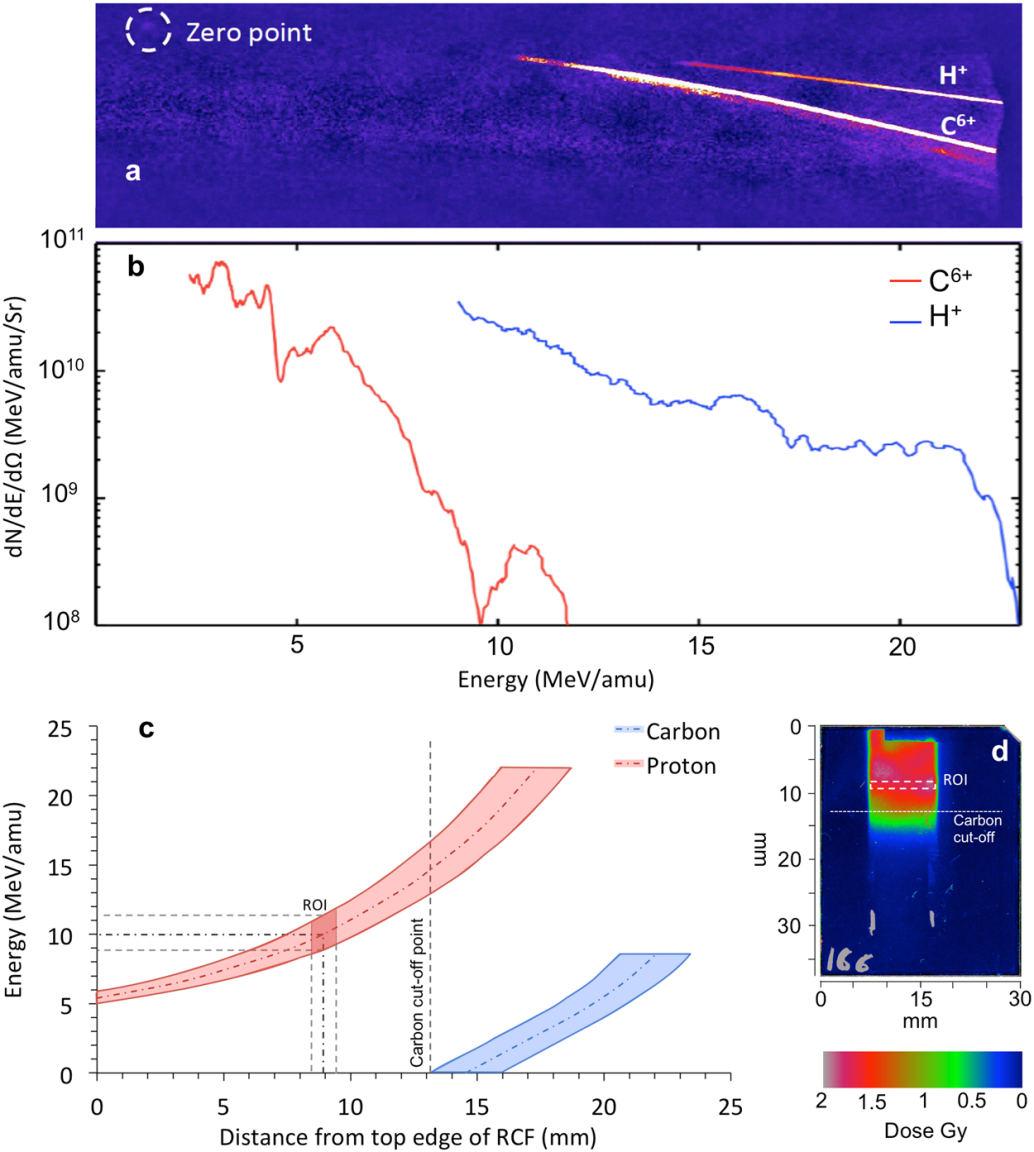
(**a**) Raw image showing the energy spectra of the ions obtained by means of Thomson Parabola Spectrometer and image plate detector. (**b**) Typical profile of the energy distribution of protons and fully ionized carbon ions. (**c**) Proton and carbon energy dispersion along the cell plane, with the origin of the x-axis at the top edge of the Gafchromic film. As shown in the figure, the carbon ions with low initial energy are filtered out for a distance of 13 mm, overlapping only with protons of energies higher than 15 MeV. The dark red quadrilateral on the protons curve represents the Region-of-Interest (ROI in the energy and distance) where the cells were selected for analysis. (**d**) The RCF film shows the typical dose distribution just behind the cells plane, and the white dashed rectangle identifies the spatial location of above-mentioned ROI on RCF.

**Figure 3:**
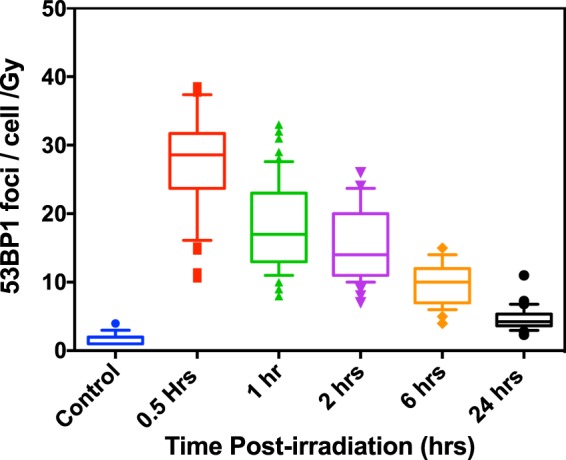
Quantitative analysis of the variations in 53BP1 foci per cell per Gy in AG01522B cells after exposure to 10 MeV (LET-4.6 keV/μm) laser-accelerated protons shown as whisker box plots generated using Prism 6 software. The lower part of the box indicate first quartile, the dividing line shows the median and top line shows the third quartile of the 53BP1 foci per cell per Gy. The lower and upper ends of the whisker indicate 10^th^ and 90^th^ percentile also indicating the outliers below 10^th^ percentile and above 90^th^ percentile. The number of cells considered for each data point ranged from 50–300 in three independent replicates.

The effect of laser-accelerated protons on the DNA DSB damage and repair was quantified by using the p53 binding protein-1 (53BP1) foci formation assay at 0.5, 1, 2, 6 and 24 hours after irradiation. The irradiation set up used in our study is shown in Fig. 1. The cells were grown on 3 μm Mylar mounted on bespoke stainless steel dishes and held perpendicular to the dispersion plane of the laser-accelerated proton beam. Beam characterization on a shot-to-shot basis was carried out via routine EBT2 Gafchromic film densitometry as shown in Fig. 2. After irradiation and immunofluorescence staining (see methods section) the cells were scored for 53BP1 foci quantification as shown in Fig. 3, in the form of Box-Whisker plots. The Box-Whisker plots show the range of foci per cell obtained at each time point. The dividing line in each box shows the median and the error bars indicate 5 and 95 percentiles of the 53BP1 foci per cell for each time point. The outliers are indicated above and below the error bars.

**Figure 4:**
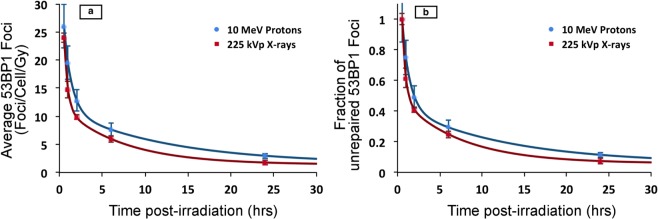
Comparative analysis of 53BP1 foci induced by 10 MeV(LET-4.6 keV/μm) laser-accelerated protons and 225 kVp X-rays. (**a**) Average 53BP1 foci calculated over time and expressed as foci per cell per Gy for both 10 MeV protons and 225 kVp X-rays. The data was fitted with a Two Phase exponential decay model, which is most commonly used for fitting the foci kinetics. For each data point cells counted ranged from 50–300 in at least 3 independent replicates. The error bars represent the standard error of the mean. (**b**) Repair kinetics of 53BP1 foci shown as percentage of the residual 53BP1 foci over time calculated by considering the average foci at 30 minutes as 100% and all the average foci per cell values were normalized with 30 minutes for each time point. Similar to figure a, the percentage of foci remaining at each time point was fitted with a two phase decay equation (Eq. 1) using the exponential non-linear regression curve fitting function of the Prism-6 software as shown in the results section.

Foci induced by the laser-accelerated protons were compared to 225 kVp X-rays induced foci and the results are shown in Fig. 4, where the experimental data were fitted by means of a biphasic (two phase) exponential decay equation, as used in Grosser *et al*.^20^:

1 $${\rm{Y}}(t)={{\rm{A}}}_{{\rm{F}}}\,\exp \,(\,-\,{{\rm{k}}}_{{\rm{F}}}\,t)+{{\rm{A}}}_{{\rm{S}}}\,\exp \,(\,-\,{{\rm{k}}}_{{\rm{S}}}\,t)+{\rm{B}}$$

where Y(*t*) is the number of foci at time *t*; A_F_ and A_S_ represent the initial induced foci related to the fast and slow kinetics, respectively; k_F_ and k_S_ are constants accounting for the fast and slow rate of repair, respectively; and B indicates background foci which are left unrepaired.

The mean foci/nucleus/Gy measured at 0.5 hours for 225 kVp X-rays and 10 MeV protons were 24 ± 1 and 26 ± 2 respectively. At the 24 hours time-point, the majority of the DNA DSB were repaired as shown by the disappearance of 53BP1 foci leading to an overall reduction in average foci number close to the background level of 1.8 ± 0.5 and 2.9 ± 0.5 respectively for X-rays and protons, while the values for controls were 1.4 ± 0.8 and 1.5 ± 0.2 respectively for X-rays and proton groups.

By fitting our data with the above model we obtained slight (non-significant) differences between the repair rates of proton-induced foci and X-ray-induced foci. The best-fit values for the constant parameters of the function were: for the fast repair kinetic, A_F_ equal to 25.9 ± 3.4 and 39.1 ± 6.1, and k_F_ equal to 1.08 ± 0.03 and 2.25 ± 0.45, respectively for protons and X-rays; for the slow repair kinetic, A_S_ equal to 9.8 ± 1.0 and 10.6 ± 0.8, and k_S_ equal to 0.080 ± 0.007 and 0.14 ± 0.03, respectively for the laser accelerated protons and X-rays. The foci background B term is assumed to be equal to the number of foci of the controls. The fast and slow half-lives were 8.7 ± 0.8 and 0.64 ± 0.02 hours for laser-accelerated protons, and 4.9 ± 1.1 and 0.31 ± 0.06 hours for X-rays. There is an observable difference in the slow and fast repair half-lives of the laser accelerated protons and X-rays induced foci, however it is uncertain whether this simply results from the higher RBE of protons compared to the X-rays or the higher dose-rate has an effect. To fully understand the implications of ultrahigh dose rate upon the DNA DSB foci kinetics, a detailed comparison with RF-accelerated protons at similar energy and LET is further warranted.

### 53BP1 foci persistence measurements

In order to obtain an insight on the complexity of DSB foci repair we further calculated the percentage of residual DNA DSB damage remaining as shown in Fig. 4b, where this is defined as the percentage of the number of radiation-induced 53BP1 foci at the time (t) with respect to the maximum number of radiation-induced foci (obtained at 0.5 hours), for the same radiation type. The foci repair kinetics in terms of percentage of foci remaining for 225 kVp X-rays and 10 MeV protons followed a two-phase exponential decay fitting, as shown in Fig. 4a and b, and as demonstrated in earlier papers for low LET radiation of 2 keV/µm and 4.59 keV/µm, respectively^21,22^.

### Sub-population radiosensitivity analysis

**Figure 5:**
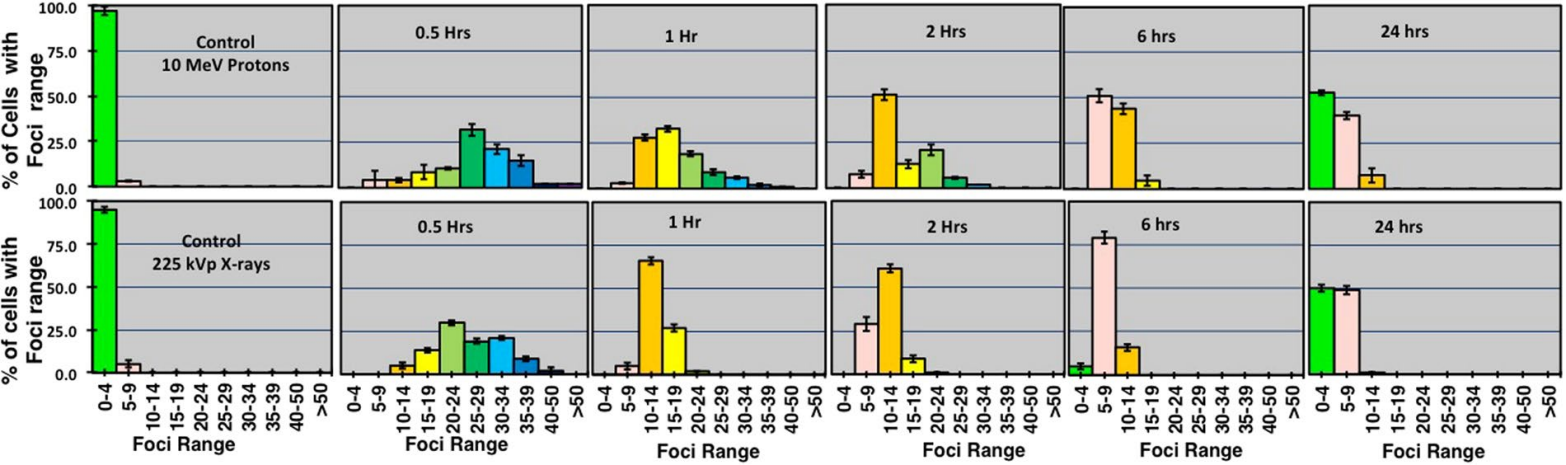
Sub-population radio-sensitivity analysis as shown through the 53BP1 foci distribution per cell. The top row shows the distribution of foci for laser-accelerated 10 MeV (LET-4.6 keV/μm) protons induced foci and bottom row for 225 kVp X-rays induced foci. The Y-axis shows the percentage of cells with foci range and X-axis in each graph shows the range of foci. For each data point all the cells scored for the average foci calculations were binned in the foci range as shown on X-axis of each graph. The error bars represent the SD of the foci per cell recorded in each group of the foci range as shown on X-axis of each sub-graph.

Additionally, the heterogeneity in the radiation response of the cells scored for foci was evaluated through sub-population radiosensitivity analysis as shown in Fig. 5. At the 6 hours time point in the X-ray irradiated group, we measured around 79% of cells showing 5–9 foci compared to the proton irradiated cells where only 50% of cells show 5–9 foci and 44% cells showed greater yields of 10–14 foci per cell. The repair of the damage (assumed following foci disappearance) could be observed for both protons and X-rays at 24 hours, where about 50% of cells showed 0–4 foci and over 45% of cells showed 5–9 foci. However in the X-ray irradiated group only 1% of cells showed 10–14 foci per cell.

### Relative Foci Induction comparisons

**Figure 6:**
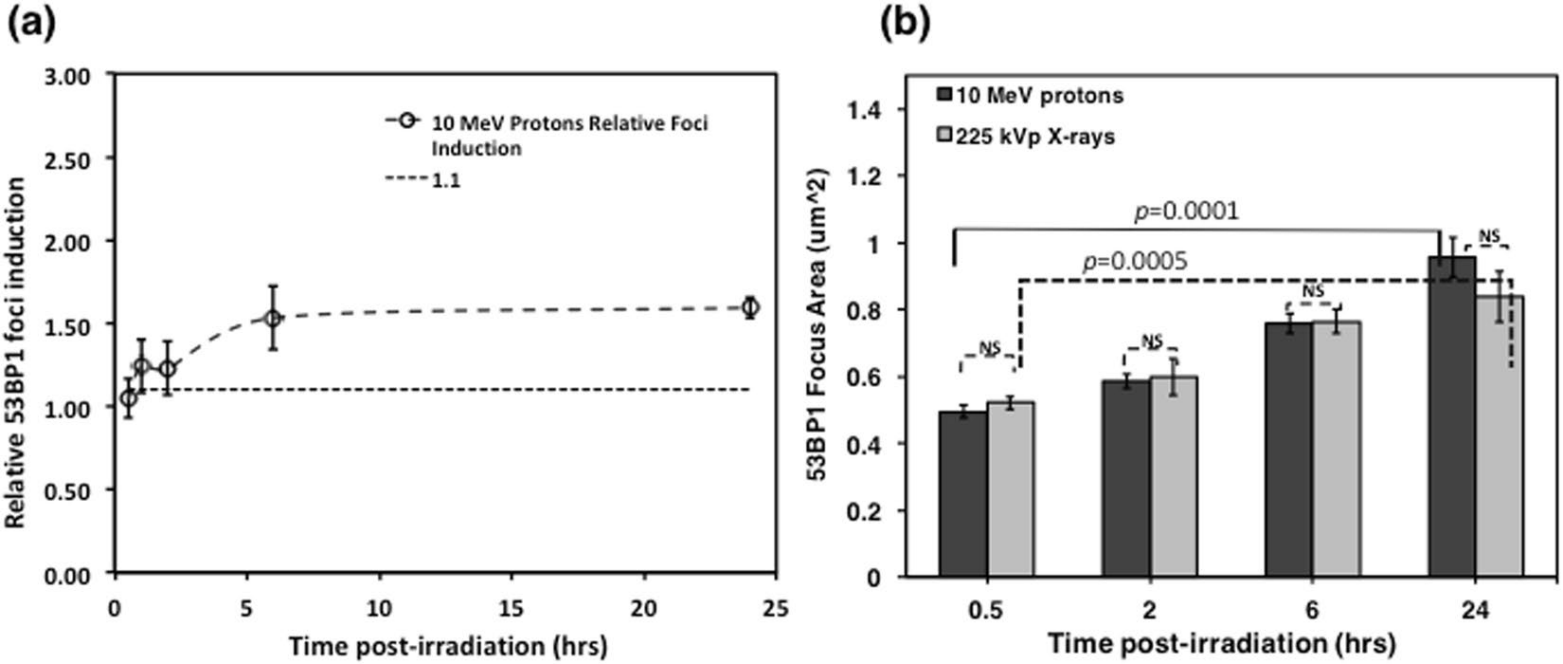
(**a**) Relative foci Induction of 10 MeV (LET-4.6 keV/μm) laser-accelerated protons to 225 kVp X-rays over 24 hours obtained by dividing the values of protons induced foci with X-rays induced foci. The dashed line represents 1.1 value based on the RBE of protons. In this paper to avoid any confusions with cell killing RBE we use the term Relative foci induction (RFI). (**b**) The size comparisons of the foci are shown in this figure here dark grey bars indicate the size of protons induced foci and light grey bars indicate the X-rays induced foci. The error bars represent standard deviations and for each data point at least 100 foci were compared values indicating the levels of statistical significance in size of the foci between 30 minutes and 24 hours; NS – non- significant.

For treatment planning optimization, a RBE value of 1.1 is typically assigned to the protons, although several investigators have shown variations in the proton RBE. While we have not calculated a cell killing RBE in this manuscript, nonetheless we compare the biological effectiveness in terms of foci induction, henceforth referred to as Relative Foci Induction or RFI, defined as the ratio of average foci per cell per Gy induced by protons to the same dose of 225 kVp X-rays in the cells. This is plotted in Fig. 6a, where a dashed line indicates the baseline of 1.1 constant value (considering a fixed effectiveness value of protons) and variations observed in the calculated RFI over time.

### Size of 53BP1 foci

We measured the size of the 53BP1 foci to gain insights on the local accumulation of 53BP1 protein in the DNA DSB domains upon damage induced by the laser-accelerated protons or the conventional 225 kVp X-rays at the 0.5, 2, 6 and 24 hours time-points post exposure. The foci sizes were analyzed using the *Analyze Particles* plugin in the ImageJ software. At least 100 foci for each data point were evaluated and the results are shown in Fig. 6b. We found a time dependent increase in the foci size from 0.5 to 24 hours, with a statistical significance of p = 0.0005 and p < 0.0001 for 225 kVp X-rays and 10 MeV protons, respectively. Overall the size of the foci induced by the laser-accelerated protons and X-rays showed a close similarity indicating the low LET nature of the laser-accelerated protons. The increased foci size observed at 24 hours, is mainly due to the presence of some of the unrepaired DSB repair foci still persisting at 24 hrs although the frequency of such cells is very low at this time point.

### Protons–induced track structure analysis

**Figure 7:**
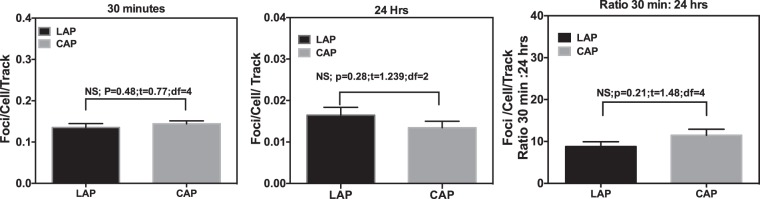
Comparative analysis of 53BP1 foci per cell per track induced by the laser-accelerated protons (LAP) and cyclotron-accelerated protons(CAP) at - (**a**) 30 minutes and (**b**) 24 hours and (**c**) the ratio of the foci per track per cell at 30 minutes to 24 hours. LET values were obtained using GEANT4 kit of Monte Carlo simulations at the various depths along the 60 MeV proton beam SOBP generated at the Douglas cyclotron of Clatterbridge Centre for Oncology, where the average LET was 4.61 keV/μm, as published by Chaudhary *et al*., IJROBP^17^. Average foci values were divided by the number of particle tracks crossing the nuclear cross section area (with radius of cell assumed to be 6.5 μm) for each time point to get foci per cell per track values. For each data point cells scored ranged from 50–300 in two independent replicates (n = 3). Statistical Significance (P < 0.05) was evaluated using Two-Tailed Unpaired T -test in Prism 6 software. P and T values for each comparison is listed on top of each graph.

Figure 7 shows the comparison of Laser accelerated protons (LAP) induced 53BP1 foci per cell per track values with cyclotron-accelerated protons (CAP) induced foci per cell per track at 30 minutes and 24 hours after irradiation. We could only compare the data at these two time points due to the availability of cyclotron accelerated protons data for similar energy at these time points only. Our results show a close correlation between the LAP and CAP induced foci per track at both time points. Further, the comparison of the ratio of foci per cell per track at 30 minutes and 24 hours in case of LAP (as shown in Fig. 7c), also matched closely to the ratio of CAP induced foci, with non-significant variations.

## Discussion

This work is broadly motivated by the prospective development of novel approaches to cancer therapy, and by the need to develop a basic understanding of the pre-clinical radiobiology in normal human cells of ion therapy at ultra high dose rates. We have used normal human skin fibroblast cell line AG01522B, a well characterized, radio-biologically relevant model system, to study DNA DSB repair dynamics following exposure to laser-accelerated protons at dose rates >10^9^ Gy/s. As the dosimetry of laser-accelerated protons is complex, slight variations in the energy and dose can make biological observations error prone, thus requiring careful consideration of all the confounding factors in dosimetry.

The dose measured by radiochromic film does not solely represent the actual dose deposited in cells and requires two corrections to adjust for the measurement limitations. The first correction takes into account the degradation of energy as the ion beam penetrates the medium: due to the active layer of the RCF being located at different positions to that of the cell monolayer, ions are required to pass through further layers of varying thickness/density before reaching the active film layer. The second correction is due to the variation of the dose response of the RCF films with ion energy and LET, as reported by Kirby *et al*.^23^. Protons delivered in the single ultra-short pulses had variable energy spectrum and for this study we used 10 MeV protons as the flux of protons at this energy was relevant to deliver dose close to 1 Gy which could be compared to the previous data we obtained for cyclotron accelerated protons as well as X-rays.

Protons, along with high LET particles, are well-reported for inducing clustered DNA lesions^24^ which can be visualized using persistent γ-H2AX or 53BP1 foci^25,26^. The foci repair kinetics curves display both the fast and slow components of the repair which predominantly describe the nature of the DSB lesion complexity. These ionizing-radiation-induced 53BP1 foci are not only mere indicators of DSB, but they are also reported as the local DNA repair centers where the damaged chromatin is processed for repair^25^. Based on the complexity of the breaks, the foci may disappear fast or persist for longer times such as upto 72 hours post ions exposure as reported by Grosser *et al*.^20^ In our experiment we observed non-significant variations in the 53BP1 foci induction and repair upto 1 hour post-irradiation with X-rays and protons, in line with previous studies^17,27^. At 24 hours post-irradiation with laser accelerated protons a slight increase in 53BP1 foci with respect to X-rays was observed which is however non-significant.

We used asynchronous cells where the cells across the distribution may not be in the same phase of cell cycle. The radiation response of an asynchronous cell culture may be heterogeneous and averaging the DNA DSB foci number may obscure any cell to cell variations as suggested by Gruel *et al*.^28^. For this reason, we measured the sub-population radiosensitivity or foci per cell distribution, as shown in Fig. 5. Variations in the foci distribution were observed as early as 30 minutes and persisted up to 24 hours. For the initial time points, the foci distribution in both the X-rays and laser-accelerated protons is Gaussian in nature (fit not shown). A clear shift in the foci distribution was observed for the later time points with an increase in the number of cells (~10%) having up to 14 foci remaining 24 hours post-irradiation in the case of laser-accelerated protons while for X-rays most of the cells at this time points had up to 9 foci.

Various groups have studied the biological effectiveness of laser-accelerated protons and calculated the relative biological effectiveness (RBE) values of laser-accelerated protons, which was reported to be 1.4 ± 0.4 and 1.3 ± 0.3 for foci induction in A549 and HeLa cells^8,10^. Schmid *et al*. have reported the micronuclei induction RBE as 1.08 ± 0.20 and 1.00 ± 0.14 for two experiments in human skin 3-D model for 20 MeV pulsed protons^27^. Belli *et al*. have reported cell killing RBE of 1.5 using 5 MeV conventional protons with an LET of ~7.6 keV/µm^29^. It should be noted that cell inactivation RBE and relative foci induction, may not be directly related to each other, as clonogenic cell death is a complex physiological process involving the multiple processes in a cell which lead to cell death. However, despite the existence of variations between the relative foci induction (RFI) and cell killing RBE, the former can still be used as a surrogate of relative effectiveness. In our study some differences in RFI were noticed for the initial time points and at 24 hours post-irradiation.

Furthermore, the amount of the residual foci remaining at 24 hours and the size of the foci showed no statistically significant difference between 225 kVp X-rays and 10 MeV protons. The foci size appeared similar between X-rays and laser-accelerated protons at 24 hours, with the size of foci increasing significantly with respect to 30 minutes size for both the X-rays and protons. This can be understood on the basis that the majority of the DSBs at earlier time-points consisted of both indirect and direct damage, while over time most of the indirect damages are repaired and the lesions formed by the direct interaction of the protons or X-rays persist longer. For foci size scoring at 24 hours, these persistent foci were the only ones contributing to the observation shown in Fig. 6b. This is supportive of the work performed by Costes *et al*.^30^, who measured changes in foci size over a 24 hour period post-irradiation and found an increase in size with time for high LET irradiation. Ibanez *et al*., also observed an increase in foci size for up to 6 hours for both lithium and protons at the Bragg peak^26^. In the experimental results presented here, although time dependent variations in the foci size were observed, these were not statistically significant which could be attributed to the similar LET values of X-rays (~2 keV/µm) and 10 MeV protons (~4.6 keV/µm).

Ion tracks are the main biophysical parameters to model radiation quality effects and predict normal tissue complication probabilities in treatment planning algorithms^31–34^. Track structure leads to clustering of DNA damage events comprising of single strand breaks, base damage, double strand breaks etc. within a few base pairs of DNA as clustered DNA damage^25,35^. Increasing LET induces more repair-refractory clustered damage further increasing the RBE of a particular radiation type^36^. Foci per track calculations are used to model the DNA DSB damage response and here we have used this approach to cross validate the dose of laser-accelerated protons delivered to the cells given that laser-accelerated proton dosimetry is still a developing area. Using conventionally accelerated proton beams we plotted the average foci per track as a function of LET for foci induction at 30 minutes and 24 hours and found a linear relationship between the LET and foci per track.

As shown in Fig. 7, the foci per track values induced by the laser-accelerated protons showed a close correlation to the data obtained with conventional cyclotron-accelerated protons for the initial or residual DSBs. The ratio of the foci per track at 30 minutes to 24 hours showed small differences between the laser-accelerated protons and conventionally accelerated protons possibly indicating the impact of ultra-high dose rate delivery on the repair of the DNA DSBs. Similar observations for the DNA DSB repair process with laser and conventionally accelerated protons were also reported, using fractionated dose delivery, by Raschke *et al*.^12^, who however did not comment on the foci per track values or the ratio of the foci per track for the initial and residual DSBs.

## Conclusion

AG01522B cells were irradiated with laser-accelerated protons in single pulses at ultra-high dose rates of the order of 10^9^ Gy/s. The induction and distribution of radiation-induced foci was measured over a 24 hour period giving a preliminary RFI for protons of 2.9 ± 0.5 at 24 hours with X-rays used as reference radiation. The residual component remaining at 24 hours and the size of the foci showed non-significant variations between 225 kVp X-rays and 10 MeV protons. Foci per track per cell analysis revealed a close correlation between the foci induced by laser-accelerated protons and cyclotron accelerated protons, broadly supporting the findings reported in previous work.

## Methods

### Cell culture and handling

AG01522B cells were maintained in α-modified Minimum Essential Medium (MEM) (Sigma Merck,) supplemented with 20% Fetal Bovine Serum (FBS) and 1% penicillin-streptomycin (Gibco, Life Technologies Carlsbad, CA, USA). All cells were incubated in 5% CO_2_ with 95% humidity at 37 °C. For this experiment 80–90% confluent T175 flasks were completely filled with warm low serum (2.5%) medium, then sealed and packed in polystyrene foam containers and shipped to the Gemini laser system at the Rutherford Appleton Laboratory, STFC, United Kingdom. Upon arrival at the facility, low serum medium was replaced with regular full growth medium and the flasks were incubated for at least 24 hours to allow cells to recover from any stress induced during transportation.

### Set up and Beam characterization

Cells were irradiated at the Rutherford Appleton Laboratory, Science and Technology Facilities Council, United Kingdom using the Gemini laser system which is able to deliver ~12 J in a single shot at a pulse duration and central wavelength of 45 femtoseconds and 0.8 µm, respectively. The laser pulse was reflected off a double plasma mirror to enhance the temporal contrast of the laser. The total throughput of the double plasma mirror was ~50%, reducing the laser pulse energy on target to about 6 J. An f/2 off-axis parabola was used to focus the linearly polarized laser pulse onto a 25 nm amorphous carbon target at normal incidence, yielding an intensity of ~6 × 10^20^ Wcm^−2^. The Ions (protons and carbon) were emitted from the rear surface of the target and spatially selected with a rectangular aperture slit (W × H = 900 µm × 400 µm) located 37 mm behind the target. A dipole magnet (100 mm long, with magnetic field strength of 0.90 T) placed behind the aperture, was used both to deflect the protons away from background radiation (X-rays, electrons) and disperse the particles at the cell plane according to their energy. The dispersed protons and carbon ions exited the vacuum chamber through a 50 µm kapton window positioned 50 mm away from the dipole output and irradiated the cells. The laser driven ion beam was characterized using a Thomson Parabola Spectrometer^37,38^, which was obtained inserting two parallel electric plates between the dipole and the kapton window. After the beam was characterized, and protons spectra were known, the electric plates were removed. A 5.5 µm thick aluminium foil was used in front of the cell dish to filter out the scattered light avoiding RCF film overexposure. Low energy carbon ions coming from the target were filtered out from the cell irradiation region of interest by the several layers of materials interposed in front of the cells monolayer plane.

### Dosimetry and Irradiation Procedure

Cells were plated 24 hours before irradiation at a density of 3 × 10^5^ per dish on 3 µm thin Mylar mounted on a customized cell dish (as shown in Fig. 1) pre-sterilized using 1 kGy dose of X-rays. Fresh medium was added to the cell monolayer and the cell dish was sealed with another 3 µm thin Mylar sheet held by a stainless steel ring, to prevent the cells from drying during the transit and irradiation procedure. For the irradiation, the cell dish was placed vertically and in air after the kapton window, with the cells at a distance of 24 mm from the kapton. The cell dish was mounted vertically on a holder which allowed to place the cell plane always at the same location with approximately ~100 µm accuracy. EBT2 radiochromic film was placed immediately behind and in contact with the 3 µm Mylar on which the cells were attached (i.e. cell plane) to measure the dose deposited in the cells. The EBT2 film was calibrated with the MC40 Cyclotron at the University of Birmingham using a monochromatic proton beam of 29 MeV.

The energy broadband TNSA beam resulted in a large region of the cells exposed to a wide range of proton energies dispersed vertically at the plane of the cells. The mean energy of protons considered for cell irradiation analysis was around 10 MeV and the corresponding LET value was about 4.6 keV/µm. Due to the short range of penetration in water of 10 MeV protons (i.e. ~1.2 mm) the culture media was removed (before each irradiation) from the dish using an automated and sterilized pump system at a slow flow rate of 0.2 ml/s, leaving only a thin film of medium on the cells^9^. Furthermore, the vertical dimension of the aperture (i.e. along the proton energy dispersion axis) used for proton energy selection and the size of the analyzed cell region of 10 mm × 1 mm (H × V) centered at 10 MeV proton energy give a proton energy range of 10 ± 1.1 MeV, that corresponds to a LET spread of 4.6 ± 0.4 keV/µm. Due to this energy spread and some inhomogeneity in the proton spatial distribution, the dose value to which cells are exposed to was obtained with an uncertainty of ≈15%.

The AG01522 cells were irradiated in a single shot with protons, delivering doses ranging from approximately 1 to 2 Gy. The time-of-flight (ToF) of protons from the target to the cell plane, the spread of proton energy along the dispersion axis due to the dipole and the size of the aperture all contribute to determining the ion pulse duration at the cell plane; in our system, 10 MeV protons delivered around 1 Gy dose in a single pulse of nanosecond duration corresponding to a dose rate of 10^9 ^Gy/s. Following irradiation, fresh cell culture media was added back to the cell dish and the cell dish was placed back into the incubator until time of fixation. Reference X-rays induced DSB damage kinetics was obtained by irradiating similar passage cells with 225 kVp X-rays using an XRAD225 X-ray cabinet (Precision X-ray Inc. N. Branford, CT, USA) fitted with a 2 mm copper filter, at the Public Health England (Chilton, Oxford) radiation facility. 53BP1 foci formation data used in Fig. 7. Data for the cyclotron accelerated protons at similar energies to the laser accelerated protons was taken from our previously published paper^17^ where the proton beam of 60 MeV was generated at the Douglas Cyclotron of the Clatterbridge Centre for Oncology, Wirral, Liverpool, UK and was degraded using PMMA beam degraders to obtain 10 MeV energy.

### 53BP1 foci formation assay

53BP1 foci formation assay was carried out following the method described in^17^ with slight modifications. Briefly, after irradiation and incubation for the stipulated time intervals, cells were washed in cold phosphate buffered saline (PBS) and fixed in a 1:1 solution of methanol and acetone (Sigma Aldrich, St Louis, MO, USA) at ~4 °C for 10 minutes. Fixed samples were stored in PBS at 4 °C until stained. For staining, cells were washed with cold PBS and permeabilized in chilled methanol, washed then blocked 10% goat serum and 0.2% triton X-100 in PBS, for 1 hour at room temperature. The cells were then probed with 53BP1 primary antibody (Novus Biologicals, Littleton, CO, USA) at a dilution of 1:1000 in blocking buffer for 1 hour at 37 °C. Subsequently the cells were washed, probed with secondary Alexa Flour® 488 conjugated secondary antibody at a dilution of 1:1000 and counterstained with prolong gold anti-fade reagent containing DAPI, after curing overnight the Mylar was incised from each dish and mounted on regular glass slides, sealed with nail paint and stored in −20 °C until scored.

### Image acquisition and Foci quantification

Images were acquired using a Carl Zeiss Axiovert 200 M Fluorescence Microscope (Carl Zeiss, Germany) with a 63X magnification oil immersion objective (numerical aperture of 1.4). The samples were imaged in 122 µm × 139 µm steps along the region exposed to 10 MeV protons and the number of 53BP1 foci were scored manually using ImageJ^39^ inside the field encompassing the 10 MeV energy. For each data point at least 50–100 cells were scored randomly in two independent sets of images for each time point. 53BP1 foci were scored in the nucleus of each cell in the field of view and the results were expressed as average foci per cell per Gy with error bars representing the standard error of the mean of two independent replicates.

## Data Availability

Data associated with the research published in this article can be accessed at: 10.17034/4283b410-4a4b-41eb-9118-e5a0ad335273.
